# 1-Allyl-3-phenyl­quinoxalin-2(1*H*)-one

**DOI:** 10.1107/S1600536811042474

**Published:** 2011-10-22

**Authors:** Hanane Benzeid, Rachid Bouhfid, Stephane Massip, Jean Michel Leger, El Mokhtar Essassi

**Affiliations:** a Laboratoire de Chimie Organique Hétérocyclique, Faculté des Sciences, Av. Ibn Battouta, BP 1014 Rabat, Morocco; b Moroccan Advanced Science, Innovation and Research (MASCIR) Foundation – INANOTECH, ENSET, Av. de l’Armée Royale, Madinat El Irfane 10100 Rabat, Morocco; c Laboratoire de Chimie Physique et Minérale, EA4138 Pharmacochimie, Université Victor Ségalen Bordeaux 2, 146 rue Léo Saignat, 33076 Bordeaux Cedex, France

## Abstract

The title compound, C_17_H_14_N_2_O, crystallizes with two mol­ecules in the asymmetric unit. The dihedral angles between the mean planes of the quinoxaline ring system and the phenyl ring in the two mol­ecules are 38.27 (10) and 37.14 (8)°. In the crystal, π-stacking along the *b* axis contributes to the crystal cohesion with an average distance between quinoxaline units of 3.397 (3) Å. Weak C—H⋯O interactions also occur.

## Related literature

For the crystal structure of 1-benzyl-3-phenyl­quinoxalin-2(1*H*)-one, see: Benzeid *et al.* (2009 ▶). For the biological activity of quinoxaline derivatives, see: Yan *et al.* (2007 ▶); Khan *et al.* (2008 ▶); Tandon *et al.* (2006 ▶).
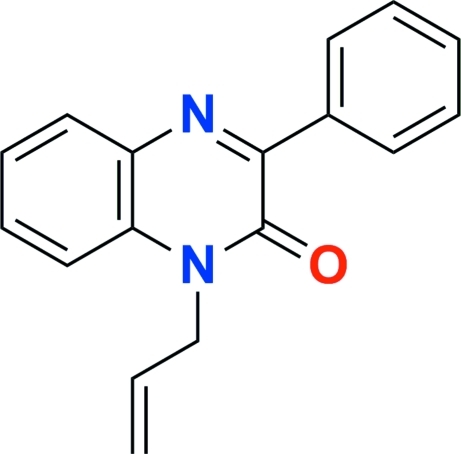


## Experimental

### 

#### Crystal data

C_17_H_14_N_2_O
*M* *_r_* = 262.30
Monoclinic, 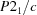

*a* = 15.123 (2) Å
*b* = 7.039 (1) Å
*c* = 26.405 (3) Å
β = 95.25 (1)°
*V* = 2799.0 (6) Å^3^
*Z* = 8
Cu *K*α radiation
μ = 0.63 mm^−1^
*T* = 296 K
0.15 × 0.15 × 0.10 mm

#### Data collection

Enraf–Nonius CAD-4 diffractometer
Absorption correction: ψ scan (North *et al.*, 1968 ▶) *T*_min_ = 0.912, *T*_max_ = 0.940
5103 measured reflections
5103 independent reflections
4037 reflections with *I* > 2σ(*I*)
2 standard reflections every 90 min  intensity decay: none

#### Refinement

*R*[*F* ^2^ > 2σ(*F* ^2^)] = 0.052
*wR*(*F* ^2^) = 0.152
*S* = 1.05
5103 reflections
362 parameters
H-atom parameters constrained
Δρ_max_ = 0.29 e Å^−3^
Δρ_min_ = −0.18 e Å^−3^

### 

Data collection: *CAD-4 Software* (Enraf–Nonius, 1989 ▶); cell refinement: *CAD-4 Software*; data reduction: *CAD-4 Software*; program(s) used to solve structure: *SHELXS97* (Sheldrick, 2008 ▶); program(s) used to refine structure: *SHELXL97* (Sheldrick, 2008 ▶); molecular graphics: *PLATON* (Spek, 2009 ▶); software used to prepare material for publication: *publCIF* (Westrip, 2010 ▶).

## Supplementary Material

Crystal structure: contains datablock(s) I, global. DOI: 10.1107/S1600536811042474/hg5102sup1.cif

Structure factors: contains datablock(s) I. DOI: 10.1107/S1600536811042474/hg5102Isup2.hkl

Supplementary material file. DOI: 10.1107/S1600536811042474/hg5102Isup3.cml

Additional supplementary materials:  crystallographic information; 3D view; checkCIF report

## Figures and Tables

**Table 1:** Hydrogen-bond geometry (Å, °)

*D*—H⋯*A*	*D*—H	H⋯*A*	*D*⋯*A*	*D*—H⋯*A*
C14—H14⋯O67^i^	0.93	2.60	3.360 (2)	140
C68—H68*B*⋯O67^ii^	0.97	2.56	3.383 (2)	143

Symmetry codes: (i) 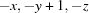; (ii) 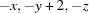.

**Fig. 1.**
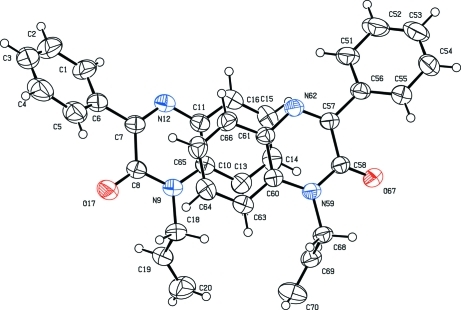
ORTEP diagram of the title molecule with the atom numbering scheme. Displacement ellipsoid are drawn at 50% probability level.

**Fig. 2.**
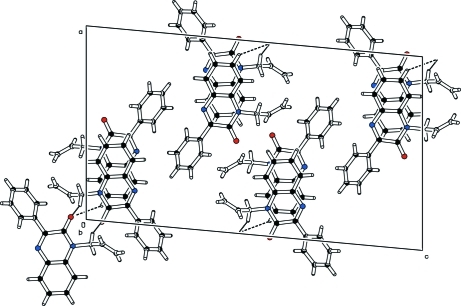
Packing diagram of the title compound viewed down the a axis. Dashed lines indicate hydrogen bonds intermolecular interactions.

## supplementary crystallographic information

### Comment

In common with other nitrogen heterocycles, quinoxalines, as well as their fused-ring bioisosteric analogs, show marked activity in many biological systems. Different quinoxaline activities are known: antibacterial (Khan *et al.* 2008), antitumoral (Yan *et al.* 2007), antiviral, and antifungal (Tandon *et al.* 2006). In this work, 1-allyl-3-phenylquinoxalin-2(1*H*)-one have been prepared by alkylation of the 3-phenylquinoxalin-2(1*H*)-one according to the following method described by (Benzeid *et al.* 2009).

The crystal structure of the title compound showed two independent molecules in the unit cell. Indeed, differences between both molecules have been noticed. Thus, torsion angles precise conformational differences as following: C1—C6—C7—N12 [36.6 (2)°] *versus* C51—C56—C57—N62 [-35.7 (2)°] for the angle between phenyl group and quinoxaline core; C8—N9—C18—C19 [90.7 (2)°] *versus* C58—N59—C68—C69 [-91.8 (2)°] for the angle between heterocycle and allyl chain. π-stacking along *b* axis participated to the crystal cohesion with an average distance between quinoxaline moieties found at 3.397 (3) Å.

### Experimental

To a solution of 3-phenylquinoxalin-2(1*H*)-one (1 g, 4.5 mmol) in dimethylformamide (20 ml), was added allylbromide (0.5 ml, 6.75 mmol), K_2_CO_3_ (1 g, 7.46 mmol) and a catalytic quantity of tetrabutylammoniumbromide. The mixture was stirred at room temperature for 12 h. The solution was filtred to remove the salts. The solvent was removed under reduced pressure. The residue was crystallized in ethanol to afford the title compound as colourless crystals. Yield: 90%, Mp: 98°C. ^1^H NMR (CDCl_3_): δ (p.p.m.) 5.02 (dd, 2H, NCH2, J=5.2 Hz); 5.29 (m, 2H,=CH2); 6.03 (m, 1H, =CH); 7.34–8.38 (m, 9H, HAr). ^13^C NMR (CDCl_3_): δ (p.p.m.) 44.8 (NCH2); 103.3 (=CH2); 130.7 (=CH); 114.1, 118.2, 123.7, 2×128.1, 2×129.6, 130.2, 130.4, 130.6, (CHAr); 132.6 136.0, 139.1, 155.0, 154.3 (Cq). Mass spectra (FAB): *M*+1= 263.

### Refinement

Carbon-bound H-atoms were placed in calculated positions (C—H 0.93 to 0.97 Å) and were included in the refinement in the riding model approximation, with *U*_iso_(H) set to 1.2–1.5Ueq(C).

### Crystal data

**Table:**

C_17_H_14_N_2_O	*F*(000) = 1104
*M**_r_* = 262.30	*D*_x_ = 1.245 Mg m^−^^3^
Monoclinic, *P*2_1_/*c*	Cu *K*α radiation, λ = 1.54180 Å
Hall symbol: -P 2ybc	Cell parameters from 25 reflections
*a* = 15.123 (2) Å	θ = 25–35°
*b* = 7.039 (1) Å	µ = 0.63 mm^−^^1^
*c* = 26.405 (3) Å	*T* = 296 K
β = 95.25 (1)°	Prism, colourless
*V* = 2799.0 (6) Å^3^	0.15 × 0.15 × 0.10 mm
*Z* = 8	

### Data collection

**Table:**

Enraf–Nonius CAD-4 diffractometer	4037 reflections with *I* > 2σ(*I*)
Radiation source: fine-focus sealed tube	*R*_int_ = 0.000
graphite	θ_max_ = 69.1°, θ_min_ = 2.9°
ω–2θ scans	*h* = −17→17
Absorption correction: ψ scan (North *et al.*, 1968)	*k* = 0→8
*T*_min_ = 0.912, *T*_max_ = 0.940	*l* = 0→31
5103 measured reflections	2 standard reflections every 90 min
5103 independent reflections	

### Refinement

**Table:**

Refinement on *F*^2^	Secondary atom site location: difference Fourier map
Least-squares matrix: full	Hydrogen site location: inferred from neighbouring sites
*R*[*F*^2^ > 2σ(*F*^2^)] = 0.052	H-atom parameters constrained
*wR*(*F*^2^) = 0.152	*w* = 1/[σ^2^(*F*_o_^2^) + (0.0862*P*)^2^ + 0.5467*P*] where *P* = (*F*_o_^2^ + 2*F*_c_^2^)/3
*S* = 1.05	(Δ/σ)_max_ < 0.001
5103 reflections	Δρ_max_ = 0.29 e Å^−^^3^
362 parameters	Δρ_min_ = −0.18 e Å^−^^3^
0 restraints	Extinction correction: *SHELXL97* (Sheldrick, 2008), Fc^*^=kFc[1+0.001xFc^2^λ^3^/sin(2θ)]^-1/4^
Primary atom site location: structure-invariant direct methods	Extinction coefficient: 0.0206 (9)

### Special details

**Table:**

Geometry. Bond distances, angles *etc*. have been calculated using the rounded fractional coordinates. All e.s.d.'s are estimated from the variances of the (full) variance-covariance matrix. The cell e.s.d.'s are taken into account in the estimation of distances, angles and torsion angles
Refinement. Refinement of *F*^2^ against ALL reflections. The weighted *R*-factor *wR* and goodness of fit *S* are based on *F*^2^, conventional *R*-factors *R* are based on *F*, with *F* set to zero for negative *F*^2^. The threshold expression of *F*^2^ > σ(*F*^2^) is used only for calculating *R*-factors(gt) *etc*. and is not relevant to the choice of reflections for refinement. *R*-factors based on *F*^2^ are statistically about twice as large as those based on *F*, and *R*- factors based on ALL data will be even larger.

### Fractional atomic coordinates and isotropic or equivalent isotropic displacement parameters (Å^2^)

**Table:**

	*x*	*y*	*z*	*U*_iso_*/*U*_eq_	
C1	0.53711 (15)	0.0487 (3)	0.19852 (7)	0.0779 (6)	
H1	0.4870	−0.0248	0.2023	0.094*	
C2	0.6140 (2)	0.0172 (5)	0.22999 (8)	0.1057 (9)	
H2	0.6159	−0.0793	0.2541	0.127*	
C3	0.68679 (18)	0.1276 (6)	0.22558 (9)	0.1188 (12)	
H3	0.7384	0.1068	0.2469	0.143*	
C4	0.68431 (17)	0.2694 (5)	0.18987 (10)	0.1122 (11)	
H4	0.7339	0.3458	0.1876	0.135*	
C5	0.60822 (14)	0.3001 (4)	0.15703 (8)	0.0856 (7)	
H5	0.6074	0.3948	0.1324	0.103*	
C6	0.53387 (13)	0.1889 (3)	0.16129 (6)	0.0626 (5)	
C7	0.44965 (12)	0.2171 (2)	0.12875 (6)	0.0516 (4)	
C8	0.45288 (11)	0.2599 (2)	0.07394 (6)	0.0511 (4)	
N9	0.37227 (9)	0.28628 (18)	0.04643 (5)	0.0474 (3)	
C10	0.29209 (11)	0.2635 (2)	0.06810 (6)	0.0468 (4)	
C11	0.29671 (11)	0.2184 (2)	0.11994 (6)	0.0494 (4)	
N12	0.37588 (10)	0.1971 (2)	0.14947 (5)	0.0538 (4)	
C13	0.20971 (12)	0.2829 (2)	0.04048 (7)	0.0563 (4)	
H13	0.2063	0.3110	0.0059	0.068*	
C14	0.13319 (12)	0.2604 (3)	0.06447 (8)	0.0627 (5)	
H14	0.0783	0.2746	0.0460	0.075*	
C15	0.13697 (13)	0.2169 (3)	0.11567 (8)	0.0666 (5)	
H15	0.0848	0.2026	0.1314	0.080*	
C16	0.21700 (13)	0.1950 (3)	0.14295 (7)	0.0614 (5)	
H16	0.2191	0.1642	0.1773	0.074*	
O17	0.52268 (8)	0.2704 (2)	0.05356 (5)	0.0683 (4)	
C18	0.37354 (12)	0.3401 (2)	−0.00747 (6)	0.0530 (4)	
H18A	0.4268	0.4135	−0.0114	0.064*	
H18B	0.3228	0.4208	−0.0171	0.064*	
C19	0.37126 (11)	0.1735 (3)	−0.04244 (6)	0.0555 (4)	
H19	0.4071	0.0698	−0.0329	0.067*	
C20	0.32244 (17)	0.1645 (4)	−0.08524 (8)	0.0908 (7)	
H20A	0.2859	0.2659	−0.0958	0.109*	
H20B	0.3239	0.0565	−0.1055	0.109*	
C51	0.02175 (13)	0.5804 (3)	0.19425 (6)	0.0662 (5)	
H51	0.0716	0.5034	0.2000	0.079*	
C52	−0.04667 (17)	0.5651 (4)	0.22526 (8)	0.0871 (7)	
H52	−0.0433	0.4758	0.2513	0.105*	
C53	−0.11933 (16)	0.6806 (4)	0.21785 (8)	0.0922 (8)	
H53	−0.1653	0.6695	0.2388	0.111*	
C54	−0.12451 (14)	0.8130 (4)	0.17954 (8)	0.0839 (7)	
H54	−0.1732	0.8939	0.1753	0.101*	
C55	−0.05793 (12)	0.8269 (3)	0.14731 (7)	0.0662 (5)	
H55	−0.0628	0.9144	0.1208	0.079*	
C56	0.01625 (11)	0.7106 (2)	0.15442 (6)	0.0520 (4)	
C57	0.09147 (11)	0.7266 (2)	0.12226 (5)	0.0458 (4)	
C58	0.07103 (11)	0.7620 (2)	0.06709 (6)	0.0465 (4)	
N59	0.14374 (9)	0.78782 (17)	0.04012 (4)	0.0450 (3)	
C60	0.22977 (11)	0.7662 (2)	0.06215 (6)	0.0446 (4)	
C61	0.24194 (11)	0.7235 (2)	0.11437 (6)	0.0463 (4)	
N62	0.17106 (9)	0.70576 (19)	0.14366 (5)	0.0487 (3)	
C63	0.30435 (12)	0.7846 (2)	0.03487 (6)	0.0529 (4)	
H63	0.2972	0.8118	0.0003	0.063*	
C64	0.38777 (12)	0.7626 (3)	0.05887 (7)	0.0590 (4)	
H64	0.4369	0.7756	0.0404	0.071*	
C65	0.40023 (12)	0.7211 (3)	0.11049 (7)	0.0609 (5)	
H65	0.4573	0.7067	0.1264	0.073*	
C66	0.32809 (12)	0.7017 (3)	0.13764 (7)	0.0569 (4)	
H66	0.3365	0.6735	0.1722	0.068*	
O67	−0.00481 (8)	0.76751 (19)	0.04607 (4)	0.0617 (3)	
C68	0.12557 (11)	0.8388 (2)	−0.01413 (5)	0.0513 (4)	
H68A	0.1725	0.9216	−0.0238	0.062*	
H68B	0.0702	0.9090	−0.0187	0.062*	
C69	0.11942 (12)	0.6708 (3)	−0.04842 (6)	0.0562 (4)	
H69	0.0846	0.5691	−0.0397	0.067*	
C70	0.15938 (17)	0.6563 (4)	−0.08966 (8)	0.0907 (7)	
H70A	0.1948	0.7554	−0.0995	0.109*	
H70B	0.1527	0.5470	−0.1094	0.109*	

### Atomic displacement parameters (Å^2^)

**Table:**

	*U*^11^	*U*^22^	*U*^33^	*U*^12^	*U*^13^	*U*^23^
C1	0.0877 (14)	0.0952 (16)	0.0482 (9)	0.0120 (12)	−0.0088 (9)	−0.0048 (10)
C2	0.109 (2)	0.149 (3)	0.0551 (12)	0.0362 (19)	−0.0155 (12)	−0.0102 (14)
C3	0.0764 (17)	0.223 (4)	0.0542 (13)	0.034 (2)	−0.0095 (11)	−0.0330 (19)
C4	0.0668 (14)	0.206 (3)	0.0642 (14)	−0.0148 (17)	0.0059 (11)	−0.0406 (19)
C5	0.0641 (13)	0.130 (2)	0.0623 (12)	−0.0137 (13)	0.0056 (9)	−0.0202 (12)
C6	0.0622 (11)	0.0808 (12)	0.0442 (9)	0.0055 (9)	0.0014 (7)	−0.0142 (8)
C7	0.0588 (10)	0.0523 (9)	0.0438 (8)	−0.0014 (7)	0.0049 (7)	−0.0055 (7)
C8	0.0554 (10)	0.0529 (9)	0.0456 (8)	−0.0028 (7)	0.0074 (7)	−0.0034 (7)
N9	0.0551 (8)	0.0459 (7)	0.0412 (7)	−0.0035 (5)	0.0052 (5)	−0.0009 (5)
C10	0.0545 (9)	0.0381 (7)	0.0480 (8)	−0.0024 (6)	0.0068 (7)	−0.0051 (6)
C11	0.0559 (9)	0.0467 (9)	0.0464 (8)	−0.0013 (7)	0.0086 (7)	−0.0042 (6)
N12	0.0617 (9)	0.0569 (8)	0.0434 (7)	−0.0006 (6)	0.0079 (6)	−0.0031 (6)
C13	0.0614 (11)	0.0494 (9)	0.0573 (10)	0.0009 (7)	0.0004 (8)	−0.0035 (7)
C14	0.0535 (10)	0.0570 (10)	0.0768 (12)	−0.0002 (8)	0.0018 (9)	−0.0115 (9)
C15	0.0569 (11)	0.0641 (11)	0.0813 (13)	−0.0060 (8)	0.0197 (9)	−0.0150 (9)
C16	0.0676 (12)	0.0638 (11)	0.0551 (9)	−0.0052 (8)	0.0186 (8)	−0.0047 (8)
O17	0.0573 (8)	0.0924 (10)	0.0568 (7)	−0.0030 (6)	0.0145 (6)	0.0012 (6)
C18	0.0629 (10)	0.0521 (9)	0.0442 (8)	−0.0048 (7)	0.0062 (7)	0.0036 (7)
C19	0.0586 (10)	0.0598 (10)	0.0492 (9)	−0.0039 (8)	0.0111 (7)	−0.0033 (7)
C20	0.1065 (18)	0.0996 (18)	0.0631 (12)	0.0072 (14)	−0.0092 (12)	−0.0218 (12)
C51	0.0788 (12)	0.0736 (12)	0.0478 (9)	−0.0004 (10)	0.0145 (8)	0.0074 (8)
C52	0.0977 (17)	0.1113 (19)	0.0560 (11)	−0.0114 (14)	0.0266 (11)	0.0098 (12)
C53	0.0757 (15)	0.148 (2)	0.0564 (12)	−0.0170 (15)	0.0267 (10)	−0.0156 (14)
C54	0.0615 (12)	0.130 (2)	0.0612 (12)	0.0114 (12)	0.0113 (9)	−0.0201 (13)
C55	0.0627 (11)	0.0847 (13)	0.0516 (9)	0.0090 (9)	0.0075 (8)	−0.0027 (9)
C56	0.0551 (9)	0.0615 (10)	0.0399 (8)	−0.0022 (7)	0.0067 (6)	−0.0039 (7)
C57	0.0553 (9)	0.0423 (8)	0.0396 (7)	0.0006 (6)	0.0045 (6)	0.0007 (6)
C58	0.0526 (9)	0.0461 (8)	0.0407 (8)	0.0027 (6)	0.0029 (6)	0.0017 (6)
N59	0.0538 (8)	0.0442 (7)	0.0370 (6)	0.0025 (5)	0.0043 (5)	0.0020 (5)
C60	0.0541 (9)	0.0364 (7)	0.0433 (8)	−0.0001 (6)	0.0039 (6)	−0.0020 (6)
C61	0.0536 (9)	0.0430 (8)	0.0420 (8)	0.0011 (6)	0.0033 (6)	−0.0015 (6)
N62	0.0544 (8)	0.0512 (7)	0.0404 (6)	0.0015 (6)	0.0030 (6)	0.0019 (5)
C63	0.0623 (10)	0.0486 (9)	0.0490 (9)	−0.0015 (7)	0.0117 (7)	−0.0010 (7)
C64	0.0536 (10)	0.0581 (10)	0.0668 (11)	−0.0042 (7)	0.0133 (8)	−0.0081 (8)
C65	0.0504 (10)	0.0629 (11)	0.0684 (11)	0.0001 (8)	−0.0004 (8)	−0.0096 (9)
C66	0.0582 (10)	0.0620 (10)	0.0491 (9)	0.0020 (8)	−0.0033 (7)	−0.0024 (7)
O67	0.0533 (7)	0.0819 (9)	0.0485 (6)	0.0040 (6)	−0.0018 (5)	0.0053 (6)
C68	0.0626 (10)	0.0515 (9)	0.0397 (8)	0.0056 (7)	0.0044 (7)	0.0064 (7)
C69	0.0617 (10)	0.0608 (10)	0.0451 (8)	0.0012 (8)	−0.0013 (7)	−0.0003 (7)
C70	0.1170 (19)	0.0961 (17)	0.0614 (12)	−0.0061 (14)	0.0214 (12)	−0.0227 (12)

### Geometric parameters (Å, °)

**Table:**

O17—C8	1.230 (2)	C16—H16	0.9303
O67—C58	1.228 (2)	C18—H18A	0.9704
N9—C8	1.373 (2)	C18—H18B	0.9698
N9—C18	1.475 (2)	C19—H19	0.9301
N9—C10	1.397 (2)	C20—H20B	0.9310
N12—C7	1.294 (2)	C20—H20A	0.9297
N12—C11	1.376 (2)	C51—C52	1.381 (3)
N59—C60	1.384 (2)	C51—C56	1.392 (2)
N59—C68	1.4779 (17)	C52—C53	1.367 (4)
N59—C58	1.376 (2)	C53—C54	1.372 (3)
N62—C57	1.290 (2)	C54—C55	1.380 (3)
N62—C61	1.384 (2)	C55—C56	1.388 (3)
C1—C2	1.384 (3)	C56—C57	1.485 (2)
C1—C6	1.391 (3)	C57—C58	1.482 (2)
C2—C3	1.361 (5)	C60—C61	1.407 (2)
C3—C4	1.371 (5)	C60—C63	1.399 (2)
C4—C5	1.393 (3)	C61—C66	1.397 (2)
C5—C6	1.383 (3)	C63—C64	1.368 (3)
C6—C7	1.483 (3)	C64—C65	1.390 (3)
C7—C8	1.483 (2)	C65—C66	1.366 (3)
C10—C11	1.401 (2)	C68—C69	1.487 (2)
C10—C13	1.392 (2)	C69—C70	1.297 (3)
C11—C16	1.408 (3)	C51—H51	0.9296
C13—C14	1.378 (3)	C52—H52	0.9296
C14—C15	1.382 (3)	C53—H53	0.9305
C15—C16	1.359 (3)	C54—H54	0.9295
C18—C19	1.491 (2)	C55—H55	0.9302
C19—C20	1.294 (3)	C63—H63	0.9293
C1—H1	0.9305	C64—H64	0.9298
C2—H2	0.9296	C65—H65	0.9302
C3—H3	0.9312	C66—H66	0.9312
C4—H4	0.9292	C68—H68A	0.9704
C5—H5	0.9306	C68—H68B	0.9703
C13—H13	0.9311	C69—H69	0.9300
C14—H14	0.9289	C70—H70A	0.9309
C15—H15	0.9301	C70—H70B	0.9296
			
C8—N9—C10	121.99 (13)	C20—C19—H19	118.04
C8—N9—C18	117.12 (14)	C19—C20—H20B	120.02
C10—N9—C18	120.89 (13)	H20A—C20—H20B	119.95
C7—N12—C11	119.18 (14)	C19—C20—H20A	120.03
C60—N59—C68	121.22 (13)	C52—C51—C56	120.21 (19)
C58—N59—C60	122.21 (12)	C51—C52—C53	120.3 (2)
C58—N59—C68	116.57 (13)	C52—C53—C54	120.0 (2)
C57—N62—C61	118.96 (13)	C53—C54—C55	120.5 (2)
C2—C1—C6	120.8 (2)	C54—C55—C56	120.08 (19)
C1—C2—C3	119.9 (3)	C51—C56—C55	118.84 (16)
C2—C3—C4	120.2 (3)	C51—C56—C57	119.09 (15)
C3—C4—C5	120.7 (3)	C55—C56—C57	122.01 (14)
C4—C5—C6	119.5 (2)	N62—C57—C56	118.27 (13)
C1—C6—C7	118.51 (18)	N62—C57—C58	123.49 (14)
C5—C6—C7	122.59 (17)	C56—C57—C58	118.23 (14)
C1—C6—C5	118.87 (19)	O67—C58—N59	121.30 (14)
C6—C7—C8	119.29 (15)	O67—C58—C57	123.47 (15)
N12—C7—C6	117.92 (14)	N59—C58—C57	115.23 (14)
N12—C7—C8	122.75 (15)	N59—C60—C61	117.97 (14)
O17—C8—C7	123.04 (15)	N59—C60—C63	123.00 (14)
N9—C8—C7	115.89 (14)	C61—C60—C63	119.03 (15)
O17—C8—N9	121.08 (15)	N62—C61—C60	121.91 (15)
N9—C10—C11	117.32 (14)	N62—C61—C66	118.89 (15)
N9—C10—C13	122.91 (15)	C60—C61—C66	119.18 (15)
C11—C10—C13	119.78 (16)	C60—C63—C64	120.26 (15)
N12—C11—C10	122.79 (15)	C63—C64—C65	120.98 (17)
N12—C11—C16	118.53 (15)	C64—C65—C66	119.51 (17)
C10—C11—C16	118.68 (15)	C61—C66—C65	121.04 (17)
C10—C13—C14	119.80 (17)	N59—C68—C69	113.13 (12)
C13—C14—C15	120.91 (18)	C68—C69—C70	124.5 (2)
C14—C15—C16	119.90 (18)	C52—C51—H51	119.87
C11—C16—C15	120.92 (17)	C56—C51—H51	119.92
N9—C18—C19	113.21 (13)	C51—C52—H52	119.86
C18—C19—C20	124.0 (2)	C53—C52—H52	119.81
C2—C1—H1	119.63	C52—C53—H53	119.98
C6—C1—H1	119.61	C54—C53—H53	119.99
C3—C2—H2	120.08	C53—C54—H54	119.74
C1—C2—H2	120.00	C55—C54—H54	119.80
C2—C3—H3	119.88	C54—C55—H55	119.93
C4—C3—H3	119.92	C56—C55—H55	119.99
C3—C4—H4	119.62	C60—C63—H63	119.88
C5—C4—H4	119.72	C64—C63—H63	119.86
C4—C5—H5	120.24	C63—C64—H64	119.55
C6—C5—H5	120.22	C65—C64—H64	119.47
C14—C13—H13	120.11	C64—C65—H65	120.18
C10—C13—H13	120.09	C66—C65—H65	120.31
C13—C14—H14	119.60	C61—C66—H66	119.52
C15—C14—H14	119.50	C65—C66—H66	119.45
C16—C15—H15	120.10	N59—C68—H68A	108.98
C14—C15—H15	119.99	N59—C68—H68B	109.03
C15—C16—H16	119.50	C69—C68—H68A	108.90
C11—C16—H16	119.58	C69—C68—H68B	108.91
N9—C18—H18B	108.89	H68A—C68—H68B	107.76
C19—C18—H18A	108.96	C68—C69—H69	117.82
N9—C18—H18A	108.92	C70—C69—H69	117.72
C19—C18—H18B	108.99	C69—C70—H70A	119.98
H18A—C18—H18B	107.74	C69—C70—H70B	120.06
C18—C19—H19	118.01	H70A—C70—H70B	119.97
			
C8—N9—C10—C11	2.1 (2)	C6—C7—C8—O17	1.2 (2)
C8—N9—C18—C19	90.75 (17)	N12—C7—C8—O17	−176.54 (15)
C10—N9—C18—C19	−89.23 (18)	N9—C10—C13—C14	−179.32 (15)
C10—N9—C8—O17	175.99 (14)	C11—C10—C13—C14	1.0 (2)
C18—N9—C8—O17	−4.0 (2)	N9—C10—C11—C16	179.83 (17)
C10—N9—C8—C7	−3.5 (2)	C13—C10—C11—N12	180.0 (5)
C18—N9—C8—C7	176.51 (12)	N9—C10—C11—N12	0.2 (2)
C18—N9—C10—C13	2.3 (2)	C13—C10—C11—C16	−0.4 (2)
C8—N9—C10—C13	−177.64 (14)	C10—C11—C16—C15	−0.5 (3)
C18—N9—C10—C11	−177.94 (13)	N12—C11—C16—C15	179.14 (18)
C11—N12—C7—C8	−0.8 (2)	C10—C13—C14—C15	−0.6 (3)
C7—N12—C11—C10	−0.8 (2)	C13—C14—C15—C16	−0.3 (3)
C11—N12—C7—C6	−178.61 (15)	C14—C15—C16—C11	0.8 (3)
C7—N12—C11—C16	179.59 (17)	N9—C18—C19—C20	136.6 (2)
C58—N59—C60—C63	177.43 (13)	C52—C51—C56—C57	178.88 (18)
C60—N59—C68—C69	87.73 (17)	C52—C51—C56—C55	1.5 (3)
C68—N59—C58—C57	−175.20 (12)	C56—C51—C52—C53	−1.5 (3)
C58—N59—C60—C61	−2.5 (2)	C51—C52—C53—C54	−0.2 (4)
C68—N59—C60—C63	−2.1 (2)	C52—C53—C54—C55	1.9 (4)
C68—N59—C60—C61	178.01 (12)	C53—C54—C55—C56	−2.0 (3)
C60—N59—C58—O67	−174.38 (14)	C54—C55—C56—C57	−177.07 (18)
C58—N59—C68—C69	−91.81 (16)	C54—C55—C56—C51	0.3 (3)
C60—N59—C58—C57	5.26 (19)	C51—C56—C57—N62	−35.7 (2)
C68—N59—C58—O67	5.2 (2)	C55—C56—C57—C58	−39.5 (2)
C57—N62—C61—C60	0.9 (2)	C51—C56—C57—C58	143.14 (15)
C61—N62—C57—C58	2.3 (2)	C55—C56—C57—N62	141.62 (17)
C61—N62—C57—C56	−178.90 (13)	C56—C57—C58—O67	−4.5 (2)
C57—N62—C61—C66	179.73 (16)	N62—C57—C58—N59	−5.3 (2)
C2—C1—C6—C5	−1.7 (3)	C56—C57—C58—N59	175.87 (12)
C6—C1—C2—C3	1.8 (4)	N62—C57—C58—O67	174.28 (15)
C2—C1—C6—C7	−179.8 (2)	C63—C60—C61—C66	0.4 (2)
C1—C2—C3—C4	−0.3 (5)	N59—C60—C61—C66	−179.73 (15)
C2—C3—C4—C5	−1.3 (5)	N59—C60—C63—C64	179.64 (16)
C3—C4—C5—C6	1.4 (4)	C61—C60—C63—C64	−0.5 (2)
C4—C5—C6—C7	178.1 (2)	C63—C60—C61—N62	179.17 (14)
C4—C5—C6—C1	0.1 (3)	N59—C60—C61—N62	−0.9 (2)
C1—C6—C7—N12	36.6 (2)	N62—C61—C66—C65	−178.88 (17)
C5—C6—C7—C8	40.7 (3)	C60—C61—C66—C65	0.0 (3)
C5—C6—C7—N12	−141.46 (19)	C60—C63—C64—C65	0.2 (3)
C1—C6—C7—C8	−141.30 (16)	C63—C64—C65—C66	0.1 (4)
C6—C7—C8—N9	−179.29 (14)	C64—C65—C66—C61	−0.2 (3)
N12—C7—C8—N9	3.0 (2)	N59—C68—C69—C70	−132.6 (2)

### Hydrogen-bond geometry (Å, °)

**Table:**

*D*—H···*A*	*D*—H	H···*A*	*D*···*A*	*D*—H···*A*
C5—H5···O17	0.93	2.50	2.921 (3)	108
C14—H14···O67^i^	0.93	2.60	3.360 (2)	140
C55—H55···O67	0.93	2.46	2.891 (2)	108
C68—H68B···O67^ii^	0.97	2.56	3.383 (2)	143

Symmetry codes: (i) −*x*, −*y*+1, −*z*; (ii) −*x*, −*y*+2, −*z*.

## References

[bb1] Benzeid, H., Saffon, N., Garrigues, B., Essassi, E. M. & Ng, S. W. (2009). *Acta Cryst.* E**65**, o2685. 10.1107/S1600536809039944 PMC2971429 21578291

[bb2] Enraf–Nonius (1989). *CAD-4 Software* Enraf–Nonius, Delft, The Netherlands.

[bb3] Khan, S. A., Saleem, K. & Khan, Z. (2008). *Eur. J. Med. Chem.* **43**, 2257–2261. 10.1016/j.ejmech.2007.09.022 18440096

[bb4] North, A. C. T., Phillips, D. C. & Mathews, F. S. (1968). *Acta Cryst.* A**24**, 351–359.

[bb5] Sheldrick, G. M. (2008). *Acta Cryst.* A**64**, 112–122. 10.1107/S0108767307043930 18156677

[bb6] Spek, A. L. (2009). *Acta Cryst.* D**65**, 148–155. 10.1107/S090744490804362X PMC2631630 19171970

[bb7] Tandon, V. K., Yadav, D. B., Maurya, H. K., Chaturvedi, A. K. & Shukla, P. K. (2006). *Bioorg. Med. Chem.* **14**, 6120–6126. 10.1016/j.bmc.2006.04.029 16806945

[bb8] Westrip, S. P. (2010). *J. Appl. Cryst.* **43**, 920–925.

[bb9] Yan, L., Liu, F. W., Dai, G. F. & Liu, H. M. (2007). *Bioorg. Med. Chem. Lett.* **17**, 609–612. 10.1016/j.bmcl.2006.11.007 17110108

